# IgG acquisition against PfEMP1 PF11_0521 domain cassette DC13, DBLβ3_D4 domain, and peptides located within these constructs in children with cerebral malaria

**DOI:** 10.1038/s41598-021-82444-5

**Published:** 2021-02-11

**Authors:** Cyril Badaut, Pimnitah Visitdesotrakul, Aurélie Chabry, Pascal Bigey, Bernard Tornyigah, Jocelyne Roman, Jules Alao Maroufou, Annick Amoussou, Blaise Serge Ayivi, Gratien Sagbo, Nicaise Tuikue Ndam, Andrew V. Oleinikov, Rachida Tahar

**Affiliations:** 1 Institut de Recherche Biomédicale des Armées, National Reference Laboratory for Arboviruses, Marseille, France; 2 Charles E. Schmidt College of Medicine, Florida Atlantic University, Boca Raton, FL 33428 USA; 3 Université de Paris, MERIT, IRD, 75006 Paris, France; 4 Université de Paris, UMR 8151 CNRS - INSERM U1022 - ENSCP, 75006 Paris, France; 5 Département de Pédiatrie, Hôpital Mère-Enfant La Lagune (CHUMEL) Cotonou, Cotonou, Benin; 6 Service de Pédiatrie, Centre Hospitalo-Universitaire, Suruléré (CHU-Suruléré, Cotonou, Benin; 7 Service de Pédiatrie, Centre National Hospitalo-Universitaire (CNHU), Cotonou, Benin; 8 Institut de Recherche Pour le Développement (IRD), UMR 261 Mère et Enfant Face Aux Infections Tropicales, Université Paris-Descartes, 4, Avenue de l’observatoire, 75270 Paris, France

**Keywords:** Immunology, Molecular biology

## Abstract

The *Plasmodium falciparum* erythrocyte-membrane-protein-1 (PF3D7_1150400/PF11_0521) contains both domain cassette DC13 and DBLβ3 domain binding to EPCR and ICAM-1 receptors, respectively. This type of PfEMP1 proteins with dual binding specificity mediate specific interactions with brain micro-vessels endothelium leading to the development of cerebral malaria (CM). Using plasma collected from children at time of hospital admission and after 30 days, we study an acquisition of IgG response to PF3D7_1150400/PF11_0521 DC13 and DBLβ3_D4 recombinant constructs, and five peptides located within these constructs, specifically in DBLα1.7_D2 and DBLβ3_D4 domains. We found significant IgG responses against the entire DC13, PF11_0521_DBLβ3_D4 domain, and peptides. The responses varied against different peptides and depended on the clinical status of children. The response was stronger at day 30, and mostly did not differ between CM and uncomplicated malaria (UM) groups. Specifically, the DBLβ3 B3-34 peptide that contains essential residues involved in the interaction between PF11_0521 DBLβ3_D4 domain and ICAM-1 receptor demonstrated significant increase in reactivity to IgG1 and IgG3 antibodies at convalescence. Further, IgG reactivity in CM group at time of admission against functionally active (ICAM-1-binding) PF11_0521 DBLβ3_D4 domain was associated with protection against severe anemia. These results support development of vaccine based on the PF3D7_1150400/PF11_0521 structures to prevent CM.

## Introduction

Cerebral malaria (CM) is a major cause of mortality in endemic countries particularly among children in Africa where 91% of the 445,000 annual deaths occurs (WHO; World Malaria Report 2018). Several studies, including recent Magnetic Resonance Imaging (MRI) observations, assumed that the cytoadhesion and accumulation of infected erythrocytes (iEs) to the brain micro-vessel endothelium are associated to the clogging and obstruction of blood circulation. This results in the disruption of blood–brain barrier (BBB) tight junctions, vascular permeability, severe edema, and micro-hemorrhages and swelling^1–4^. It has been shown that iEs adhesion to endothelium is mediated by PfEMP1 protein family, the major clonally-expressed variant surface molecules involved in the malaria pathogenesis and antigenic variation^5–7^. The high polymorphism of these proteins enable iEs to modify their antigenic and binding properties allowing them to escape the host immune system^8^. Nevertheless, in endemic areas, individuals gradually acquire a protective immunity to various parasite-blood stage antigens including PfEMP-1 after limited number of malaria episodes, preventing them from severe and cerebral malaria complications^9–11^.

PfEMP1 proteins are encoded by ~ 60 members of the *var* gene family, which undergo transcriptional switch allowing the parasites to express a single clonal variant protein at a time^12–14^. These proteins are composed of multiple Duffy binding like (DBL) and cysteine-rich interdomain region (CIDR) domains, which are classified according to their sequence into several types (α, β, γ, σ, ε, ζ) and their numbered subtypes^7^. Sequence analysis of *var* genes from seven genomes led to the identification of twenty novel conserved structural units, so called domain cassettes (DC), each composed of several specific domain types^15^. Recent studies showed that parasite isolates causing severe and cerebral malaria transcribed preferentially *var* genes with DC 08, 13 and 04^16–19^. These DC have been demonstrated to bind to the endothelial protein C receptor (EPCR) through their CIDRα1.1/CIDRα1.4 domains^20^. The presence of parasites expressing PfEMP1s with dual binding capacity to ICAM-1 and EPCR was shown in CM isolates^19,21^. In addition, antibodies against recombinant ICAM-1-binding DBLβ3 domain, which are common in adults^22^, have been associated with protection against severe and moderately severe malaria hospitalizations^23^ and with a reduced risk of high-density clinical and severe malaria^24^. Therefore, these findings emphasized the importance of these specific sequence motifs/domains in the pathogenesis of severe and cerebral malaria and raised the question on their involvement in triggering the acquisition of protective antibodies in CM. In this study, we report on specificity of antibody immune response to PF3D7_1150400/PF11_0521 DC13 recombinant protein, to full-length functional (ICAM-1 binding) domain DBLβ3_D4, and to peptide motifs that aligned to the DBL α 1.7_D2 and DBLβ3_D4 domains of this protein in children with CM.

## Material and methods

### Patient’s samples

Plasmas samples were collected from children consulting at Centre hospitalier universitaire Mère-enfant de la Lagune (CHUMEL), Centre National Hospitalier Universitaire Hubert Koutoucou Mega (CNHU-HKM), or to the Centre Hospitalier Universitaire of Suru-Léré CHU Suru-Léré, Cotonou, Benin, for malaria during the period of high malaria transmission (June–September 2012 and May–July 2013, respectively). Informed consent was obtained from a parent and/or legal guardian for all children enrolled in the study.

Children of 6 years old or less were recruited in the study if they presented positive rapid diagnostic test for malaria (DiaQuick Malaria P. falciparum Cassette, Dialab; Hondastrasse, Austria) confirmed microscopically by Giemsa-stained thick blood smears, and meet the WHO clinical malaria definition criteria. CM group was defined as a microscopically confirmed *P. falciparum* infection and a Blantyre coma score ≤ 2, with the exclusion of any other causes of coma. Uncomplicated malaria group (UM) have *P. falciparum* parasitemia infection along with fever, headache, or myalgia without signs of life-threatening malaria and evidence of vital organ dysfunction, as defined by the World Health Organization (WHO; 2000). Both ELISA experiments using PF11_0521-DC13 recombinant protein and peptides as well as IgG reactivity to the DBLβ3_D4 immobilized on BioPlex beads were performed on children with CM from the same cohort.

Samples of 2–4 ml of blood were collected into citrate phosphate dextrose adenine-containing tubes from all children whose parents signed the informed consent. All patients were treated according to the guidelines established by the Beninese Ministry of Health. Children with severe cases were treated with 120 mg quinine perfusion.

Positive controls: the first positive control, included in triplicate to each ELISA plate, consisted of a pool of 20 plasmas from highly responsive individuals to MSP3 and GLURP (R0; R1 and RII) antigens. These plasmas were collected from adult males in Senegal. The second positive control were plasmas from individual children aged between 4 and 5 years old living in endemic malaria transmission area of the suburb of Yaoundé, Cameroun, tested individually in triplicate. These children were asymptomatic carriers of *P. falciparum* mono infection.

Negative controls consisted of a pool of 20 plasma samples from European adult blood donors who had never traveled to malaria endemic areas.

The study protocol was reviewed and approved by the Research Institute of Applied Biomedical Sciences ethics committee (ISBA-CER), Cotonou, Benin (No 006/CER/ISBA/12 and No 21/CER/ISBA/13) and the Cameroonian National Ethics Committee as well as the Cameroonian Ministry of Public Health (authorization No 028/CNE/DNM/07). All the methods used in this study were performed according to the relevant guidelines and regulations of the ISBA-CER and the Cameroonian Ministry of Public Health.

### Peptides design

The number, type and boundaries of each DBL and CIDR domains of PF11_0521/PF3D7 1,150,400 protein were determined by the site http://www.cbs.dtu.dk/services/VarDom/. Each DBL or CIDR sequence was isolated and homologous or paralogous sequences were found by searching for the nearest matching sequences at the site: http://blast.ncbi.nlm.nih.gov/ Blast.cgi. The DBL and CIDR sequences with the highest score were selected and aligned using the site: http://multalin.toulouse.inra.fr/multalin/. The solved (or predicted using https://toolkit.tuebingen.mpg.de/tools/hhpred) structures of the DBLs and CIDRs of interest were analyzed. The sequences of each of the corresponding variable regions for each DBL and CIDR domain were aligned. These analyses allowed us to determine constant and variable regions where only a few consensus sequences were observed, to be considered as sequences subjected to a selection pressure due to the host immune system escapement, as already described for the DBL6ε domain^25,26^. The absence of local homology to other existing proteins other than PfEMP1 was verified. DBLs alignments highlighting conserved and variable sequences are shown in supplementary Fig. 1 and 2. The solvent accessibility of the amino acids present in the selected variable sequences was identified using 3 D-structure. Two peptides from the PF11_0521 DBLα1.7_D2 domain (also termed in the literature as DBL1α1.7), and three peptides from the PF11_0521 DBLβ3_D4 domain (also termed in the literature as DBL2β3) were synthetized and tested for seroreactivity. The names, length, and location of the peptides are presented in Supplementary Table 1. The aligned sequences are shown in Supplementary Fig. 2. The PF11_0521 domain organizational structure and known binding receptor specificity of domains, as well as location of the peptides used in this work in the 3D structures of relevant domains are shown in Supplementary Fig. 3. To measure the exposure level of children to malaria, we also synthetized and tested peptides that belong to the C terminal domain of MSA3-based vaccine recombinant protein, containing three B cell epitope regions (a, b, and c), and three T helper cell epitopes, which were previously shown to reflect malaria exposure by the IgG response to this protein^27^.

**Figure 1:**
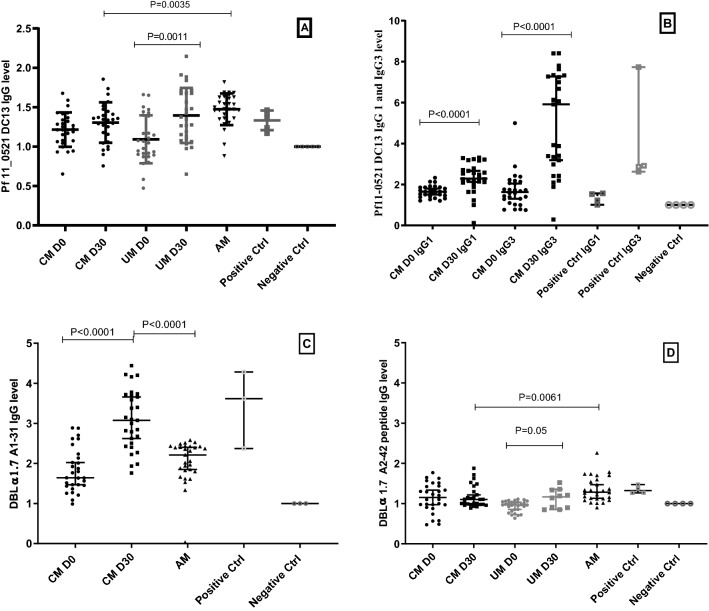
(**A**) IgG response against Pf11_0521DC13 in children with cerebral malaria (CM) at Day 0 black circles, at Day 30 black squares, in children with uncomplicated malaria (UM) at day 0 Grey circles and at day 30 Grey squares. (**B**) IgG1 and IgG3 response of CM children at Day 0 black circles and at Day 30 black squares. (**C**) IgG response against the DBLα A1-31 peptide in CM children and (**D**) IgG response of CM and UM children to the DBLα A2-42 peptide. In all graphs black triangles, empty square, empty circle highlights respectively Asymptomatic children (AM) the positive control and the negative control.

**Figure 2:**
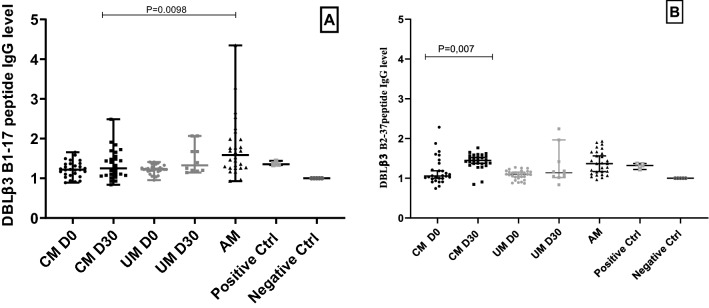
(**A**, IgG response against DBLβ3 B1-17 in children with CM at Day 0 black circles, at Day 30 black squares and children with UM at Day 0 Grey circles and at Day 30 Grey squares. (**B**) IgG response against DBLβ3 B2-37 peptides in children with CM at Day 0 black circles and at Day 30 black squares and in children with UM gray circles at Day 0 and gray squares at Day 30.

### DC 13 recombinant protein expression and purification

The DNA sequences coding for PfEMP1 PF11_0521 DC13 of Pf3D7_1150400/PF11_0521 gene composed of DBLα1.7_D2 – CIDRα 1.4_D3 domain tandem (these domains were also named in the literature as DBL1α1.7 and CIDR1α 1.4) were chemically synthesized with codon optimization for *E. coli* expression. A sequence coding for a 6His-tag was added to the 3′-terminus of this construct. Encoded 727 amino acid residues (85.03 kDa) DC13-6His construct was expressed in *E coli* and purified in denaturing conditions in 8 M urea, and then refolded by rapid dilution to 2 M urea. Wells of ELISA 96-well, high protein-binding plates (Nunc MaxiSorp Thermofisher Courtaboeuf Cedex (Villebon-sur-Yvette) were coated with these proteins at 5 µg/mL concentration. All the peptides and the DC13 recombinant protein were synthetized by ProteoGenix SAS (67300 Schiltigheim, France).

### Assessments of IgG reactivity levels against entire PF11_0521 DC13 construct, and synthetic peptides located in domain DBLα 1.7_D2 of this construct and in neighboring downstream domain PF11_0521 DBLβ3_D4.

An enzyme-linked immunosorbent assay (ELISA) to measure DC13- and peptide-specific IgG in plasma from Beninese children with cerebral malaria (CM) or uncomplicated malaria (UM) was performed in a blinded manner. Nunc MaxiSorp microtiter plates were coated with 50 µl of each peptide or recombinant protein at a concentration of 5 µg/ml in PBS pH 7,4 (Gibco, life technologies) and incubated overnight at room temperature. The wells were blocked for one hour with 100 µl of blocking solution PBS-Bovine Serum Albumin (BSA) 4%, Sigma. The plates were washed three times with 200 µl of PBS-0.05% Tween 20 (PBST). Fifty µl of Plasma samples diluted 1:100 in PBST were incubated in triplicate at room temperature for 1 h. After washing, bound antibodies were detected with horseradish peroxidase (HRP)-conjugated anti-human IgG (1 h incubation at 1:10,000 dilution) followed by washing and addition of 50 µl of 3,3′,5,5′-Tétraméthylbenzidine (TMB substrate, Sigma). The reaction was stopped by addition of 50 µl sulfuric acid to 0.2 M. IgG1 and IgG3 isotypes were also assessed against the entire DC13 from PF11_0521 protein (composed of DBLα1.7_D2-CIDRα1.4_D3 domains) in children with CM at admission (day 0) and at convalescence (day 30) and against the peptide DBLβ3 B3-34 located in the PF11_0521 DBLβ3_D4 domain in children with CM and those with UM collected at day 0 and day 30 of admission.

The optical density (OD) values were measured at 450 nm using a Biochrom Asys UVM 340 reader and corrected for background by subtraction of OD values obtained in BSA-blocked wells. Antibody reactivity was expressed in Optical Density ratio (ODr) units calculated by the mean OD of samples divided by the mean OD of the pooled negative control. All means were calculated from the triplicate measurements. Each ELISA plate included a pool of 20 plasmas from malaria non-immune people in triplicate (Negative Control), which was used to determine the precise cut-off for positive and non-reactive samples.

Median values of ODr were plotted together with CI95 using Prism v6 software (GraphPad Software, Inc., San Diego, CA, USA). The Median values and the 25% and 75% interquartile values are presented in supplementary Table 2. The non-parametric Mann–Whitney U test was used to compare sample groups. The level of statistical significance was set at 0.05.

The same pool of positive and negative plasma controls was systematically included in each ELISA plate throughout the study.

### Antibody reactivity to the PF11_0521 DBLβ3_D4 domain

The PF11_0521 DBLβ3_D4 domain and the control construct HAE (a product of expression from vector plasmid pHisAdEx without insertion of PfEMP1 construct, described below) were expressed in COS7 cells and coupled to BioRad BioPlex beads as described in^22,28^. This domain expressed in mammalian system and immobilized on BioPlex beads is correctly folded, which was assumed from its strong binding activity toward ICAM-1, also confirmed for this work (Supplementary Fig. 4). Briefly, HAE control construct contains all the parts present in DBL domain construct, but, instead of the PF11_0521 DBLβ3_D4 domain, it has irrelevant peptide of 37 amino acid residues. For each plasma sample (children or non-immune control plasma samples), the reactivity obtained against HAE control construct was subtracted from that of the DBLβ3_D4 domain (measured in the same well, all samples tested in duplicate). Then average reactivity of non-immune controls plus 2 standard deviations was subtracted from average reactivity of each children plasma sample. These data, expressed in fluorescence arbitrary units, were stratified by high and low reactivity level (above and below the median of the entire set of samples, accordingly), and associated to hemoglobin (Hb) and parasitemia (PE) levels in corresponding patients. We also measured the reactivity of plasma against AMA-1 construct, representing AMA-1 extracellular part, expressed in the same system^29^, as this protein might reflect the level of exposure of children to *P. falciparum* malaria, and analyzed data similarly by stratification to high and low anti-AMA IgG levels. Samples from children with CM at admission (day 0) were tested in these experiments. Normal distribution in all sets of data was tested by three tests (D'Agostino & Pearson, Shapiro–Wilk, and Kolmogorov–Smirnov) using GraphPad Prizm software. Statistical significance (P < 0.05) was determined by t-tests for normally distributed data and by Mann–Whitney tests for non-parametric distributions.

## Results

The clinical and biological features of children included in the study are summarized in Table 1. Briefly, we included 63 children with cerebral malaria (CM) and 35 with uncomplicated malaria (UM). There was no noticeable difference in the male to female sex ratio, temperature, and parasitemia. However, we found a statistically significant difference in the age, hemoglobin level, Blantyre score, and number of deaths. Children with CM were younger had lower hemoglobin level with Blantyre score ≤ 2 and 21 among them died.

**Table 1:** Characteristics of children enrolled in the study.

	Children with cerebral malaria (N = 63)	Children with uncomplicated malaria (N = 35)	P value
Sex ratio F/M	26/ 37	13/22	
Age month	44.5 [7–72]	33 [10–58]	0.0235
Temperature	38.2 [35.5–40.9]	38.75 [36–40.7]	0.3458
Parasitaemia P/µl median [range]	71,912.44 [1280–757333]	50,721.5 [76–160675]	0.219
Hemoglobin g/dl median [range]	5.6 [2.10–13.87]	8.75 [6.1–11.5]	< 0.0001
Glycemia g/l median [range]	0.95 [0–3.81]	ND	
Blantyre score median [range]	2 [0–2]	5	< 0.0001
Number of deaths	21	None	< 0.0001

### Induction of IgG response to group A sub-type DBL α and β domains

#### Antibody response to PF11_0521 DBLα1.7_D2-CIDRᾳ1.4_D3 (PF11_0521 DC13) recombinant protein

Our results show differential profiles of antibody response of children against the peptides and the whole Pf11_0521 DC13 (DBLα1.7_D2-CIDRα1.4_D3) recombinant protein. This response varies according to the tested molecules and to the clinical and immunological status of children. The median reactivity and 25%-75% interquartile ranges for each the DC13 and peptides are presented in Supplementary Materials Table 2. As expected, the level of response was usually higher in children at day 30 after the malaria episode (convalescence) than at day 0 at admission.

The antibody response against PF11_0521 DC13 recombinant protein was induced in 56%, 70%, 30%, 63%, 93% of children with CM day 0, CM day 30, UM day 0, UM day 30, and children with asymptomatic parasitemia (AM group), respectively, based on ODr median values. Medians and their 25–75% interquartile ranges (in parentheses) were 1.21 (1.07–1.35); 1.34 (1.11–1.46); 1.06 (0.89–1.39); 1.36 (1.10–1.68); 1.48 (1.36–1.63), respectively. There was no statistically significant difference in the response for the plasma collected at days 0 and 30 in the CM group. However, this difference was significant in the UM group (P = 0.001). Similarly, there was no statistically significant difference either between CM and UM groups at day 0 or CM and UM groups at day 30. The IgG level of AM group was higher than that of CM at day 30 and the difference was statistically significant (P = 0.0035) (Fig. 1A).

In the CM group we also assessed IgG1 and IgG3 antibody isotypes. We found that 100% of CM day 0 and 93% CM day 30 individuals had an IgG1 response with ODr median values of 1.64 (1.43–1.86) and 2.289 (2.047–2.768), respectively. At the same time, 75% of CM day 0 and 96% of CM day 30 had an IgG3 response with ODr median values of 1.63 (1.143–2.121) and 5.914 (2.98–7.33) CM day 30, respectively. Both IgG1 and IgG3 responses to the PF11_0521 DC13 in CM group at day 30 were higher than that at day 0 with statistical significance (P < 0.0001) (Fig. 1 B).

### Antibody response to peptides located in PF11_0521 domain DBLα1.7_D2

The IgG response against PF11_0521 DC13 peptide DBLα1 A1-31 located in the DBLα1.7_D2 domain was above the negative control in all but one samples with ODr median values (interquartile range) of 1.64 (1.44–2.19), 3.074 (2.467–3.71), and 2.21 (1.81–2.42) in CM Day 0, CM Day 30, and AM groups, respectively. The level of this response was higher in the CM day 30 group compared to CM day 0 with a statistically significant difference (P < 0.0001). Unexpectedly, the response of the AM group was lower than that of CM group at day 30 with statistically significant difference (P < 0.0001) (Fig. 1C).

The second peptide located in the PF11_0521 DBLα1.7 domain and named DBLα1 A2–42 was also assessed in CM and UM group and demonstrated reactivity in 57%; 81%; 37%; 70% and 67% of children in CM day 0; CM day 30; UM day 0; UM day 30, and AM groups, respectively. Medians and their 25%-75% interquartile ranges were 1.15 (0.92–1.43); 1.10 (0.98–1.30); 0.97 (0.83–1.04); 1.17 (0.88–1.34); 1.29 (1.12–1.58), respectively. There was no noticeable difference in the IgG response in the CM group between day 0 and day 30. However, IgG response in children with UM was statistically significant for samples collected at days 0 and 30 (P = 0.05). As expected, the IgG level was higher in the AM group compared to CM group (P = 0.0061) (Fig. 1 D).

### Antibody response to peptides located in PF11_0521 domain DBLβ3_D4

IgG response to the DBLβ3 B1-17 peptide located in the DBLβ3_D4 domain was very low in CM and UM groups. The ODr values were 1.22 (1.08–1.36); 1.25 (1.07–1.55); 1.22 (1.17–1.27); 1.32 (1.20–1.77); and 1.59 (1.26–1.98) for CM day 0; CM Day 30; UM day 0, UM 30 and AM group, respectively, with 64%, 51%, 62%, 80% and 92% of responders among these groups. This response seems to be similar within and between the CM and UM groups. However, the AM group IgG response was higher than that of CM group at day 30 and this difference was significant (P = 0.0098). (Fig. 2 A).

The DBLβ3 B2-37 peptide, which is also located in the DBLβ3_D4 domain induced antibody response in 28%; 96%; 10%; 30% and 64% of children with CM at day 0, CM at day 30, UM at day 0, UM day at 30, and AM group, respectively. The ODr medians were 1.06 (0.99–1.33); 1.45 (1.37–1.56); 1.10 (1.01–1.17); 1.14 (1.03–1.56); 1.37 (1.12–1.65), respectively, in these groups. The response of CM day 30 was higher than that of CM day 0 with statistically significant difference (P = 0.007). However, there was no difference in the IgG level between AM group and that of children with CM day 30 (Fig. 2 B).

**Figure 3:**
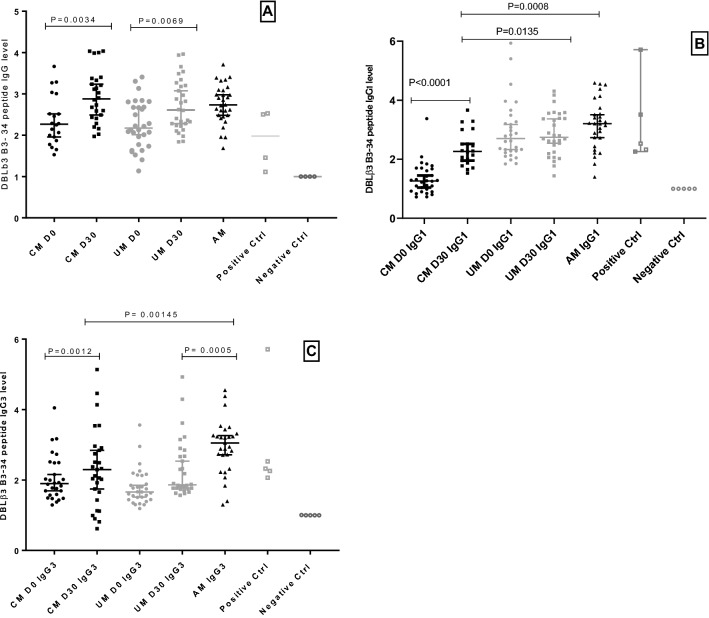
(**A**) IgG response against DBLβ3 B3-34 peptide in children with CM at Day 0 black circles at Day 30 black squares, in children with UM at Day 0 Grey circles and at Day 30 Grey squares. (**B**) and (**C**) is the IgG1 and IgG3 response against DBLβ3 B3-34 peptide in CM children Day 0 black circles, Day 30 black squares and UM children Day 0 Grey circles and Day 30 Grey squares.

The antibody response to peptide DBLβ3 B3-34 located in DBLβ3_D4 was elicited in 100% individuals of all groups with relatively higher ODr medians (2.27 (1.89–2.89); 2.88 (2.43–3.34); 2.17 (1.76–2.71); 2.61 (2.23–3.22) and 2.74 (2.47–3.09) in children with CM day 0 CM day 30, children with UM day 0 UM day 30, and AM group, respectively). The difference in the antibody response was higher at day 30 vs. day 0 with statistical significance in both UM (P = 0.0069) and CM clinical groups (P = 0.0092) (Fig. 3A). In similarity to DBLβ3 B2-37, we did not notice a difference in the response level between CM group at day 30 and the AM group.

In addition, IgG1 antibody response for this peptide demonstrated 100% responders in all groups except CM at day 0, for which we observed 40% of responders. The ODr medians and interquartile values were 1.26 (0.91–1.68), 2.27 (1.89–2.89), 2.70 (2.27–3.32), 2.74 (2.23–3.43) and 3.21 (2.58–3.63) in CM day 0, CM day 30, UM day 0, UM day30, and AM groups, respectively. It is clear that IgG1 response was higher at day 30 than at day 0 in CM group with statistically significant difference (P < 0.0001). This response was higher in UM day 30 group compared to CM day 30 group with statistical significance (P = 0.0135) and was not significantly different from that of the AM group. However, the IgG1 response of CM group was lower than that of AM group with statistically significant difference (P = 0.0008) (Fig. 3B).

Similarly, 100% of children with UM day 30, CM day 0, and AM individuals had an IgG3 response, while 96% of UM day 0 and 81% of CM day 30 individual responded. The ODr medians were 1.89 (1.58–2.49), 2.29 (1.54–2.94), 1.66 (1.44–2.13), 1.86 (1.76–2.72), and 3.05 (2.51–3.37) for CM day 0, CM day 30, UM day 0, UM day 30, and AM control, respectively. The response was higher at day 30 than at day 0 for both CM and UM groups, but the difference was statistically significant only in CM group (P = 0.0012). The AM group IgG3 response was higher than that of both UM and CM groups at day 30 and the difference was statistically significant (P = 0.0005) and (P = 0.0145), respectively (Fig. 3C).

### IgG response in children with CM to peptides from Merozoite Surface Antigen 3 and to extra-cellular part of AMA-1 antigen

**Figure 4:**
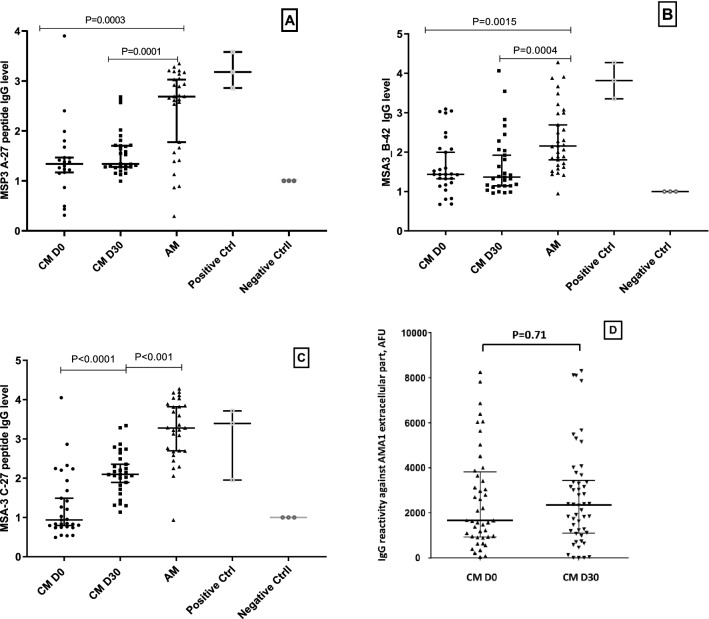
IgG response against MSA3 A-27 (**A**), MSA3 B-42 (**B**), MSA3 C-27 (**C**) peptides and against extracellular part of AMA-1 antigen (**D**) in children with CM at Day 0 and Day 30. Data for parts **A**, **B**, and **C** were obtained by ELISA. Day 0—black circles, Day 30—black squares, AM children black triangles. Empty squares are the positive control and empty circles are the negative control. Data for part D was obtained using BioPlex bead-immobilized construct. P value by Mann–Whitney test.

The IgG response in children against peptide MSA-3 A-27 shows reactivity of 73% of children with CM at day 0; 80% at day 30, and 93% of AM children. ODr medians and interquartile range were 1.34 (1.16–1.62), 1.34 (1.27–1.71), 2.69(1.61–3.09), respectively. There was no difference in the level of the response between CM at admission and convalescence. Similarly, 66%, 62%, 94% of CM at admission, CM at day 30, and AM individuals responded to peptide MSA3 B-42 with ODr median values of 1.44 (1.18–2.09), 1.37 (1.14–2.05), 2.15 (1.68–2.99), respectively. Another peptide, MSA3 C-27, reacted with only 44% of children with CM at admission, while 96% of children with CM at convalescence and children with AM were reactive. Antibody reactivity ODr median values were 0.94 (0.76–1.81) for children with CM at admission, 2.1 (1.77–2.57) at convalescence, and 3.28 (2.68–3.86) for AM group, with statistically significant difference in the groups of children with CM at days 0 and 30 (P = 0.0001). As expected, the level of the AM group IgG response against the three MSA3 peptides was higher than that of CM group at day 30 with statistically significant difference of P = 0.001 for MSA3 A-27 and MSA3 C27 peptides and P = 0.0004 for MSA3 B-42 (Fig. 4A,B,C).

We also assessed reactivity of plasma from children with CM collected at day 0 and day 30 against extra-cellular part of AMA-1 antigen, immobilized on BioPlex beads. The results presented in Fig. 4 D demonstrate no significant difference (p = 0.71), indicating similar malaria exposure level for these two group of samples.

### CM children reactivity to PF11_0521 DBLβ3_D4 domain at the time of admission

**Figure 5:**
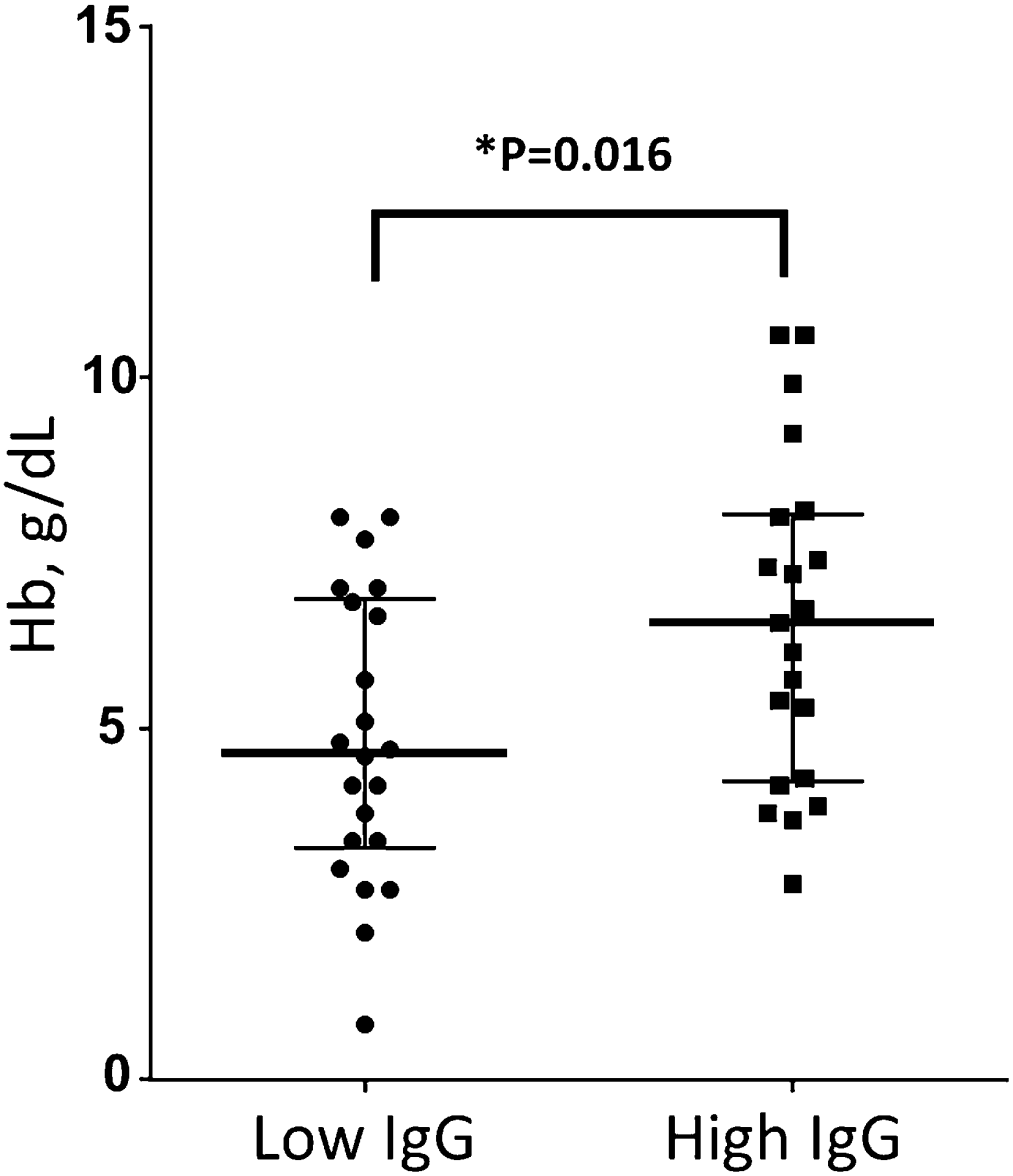
Hb levels in CM children at admission stratified by high (above median) and low (below median) IgG levels against PfEMP1 domain PF11_0521 DBLb3_D4. Red bars indicate medians and interquartile range. Data points in both sets distributed normally. P value by unpaired t-test.

**Figure 6:**
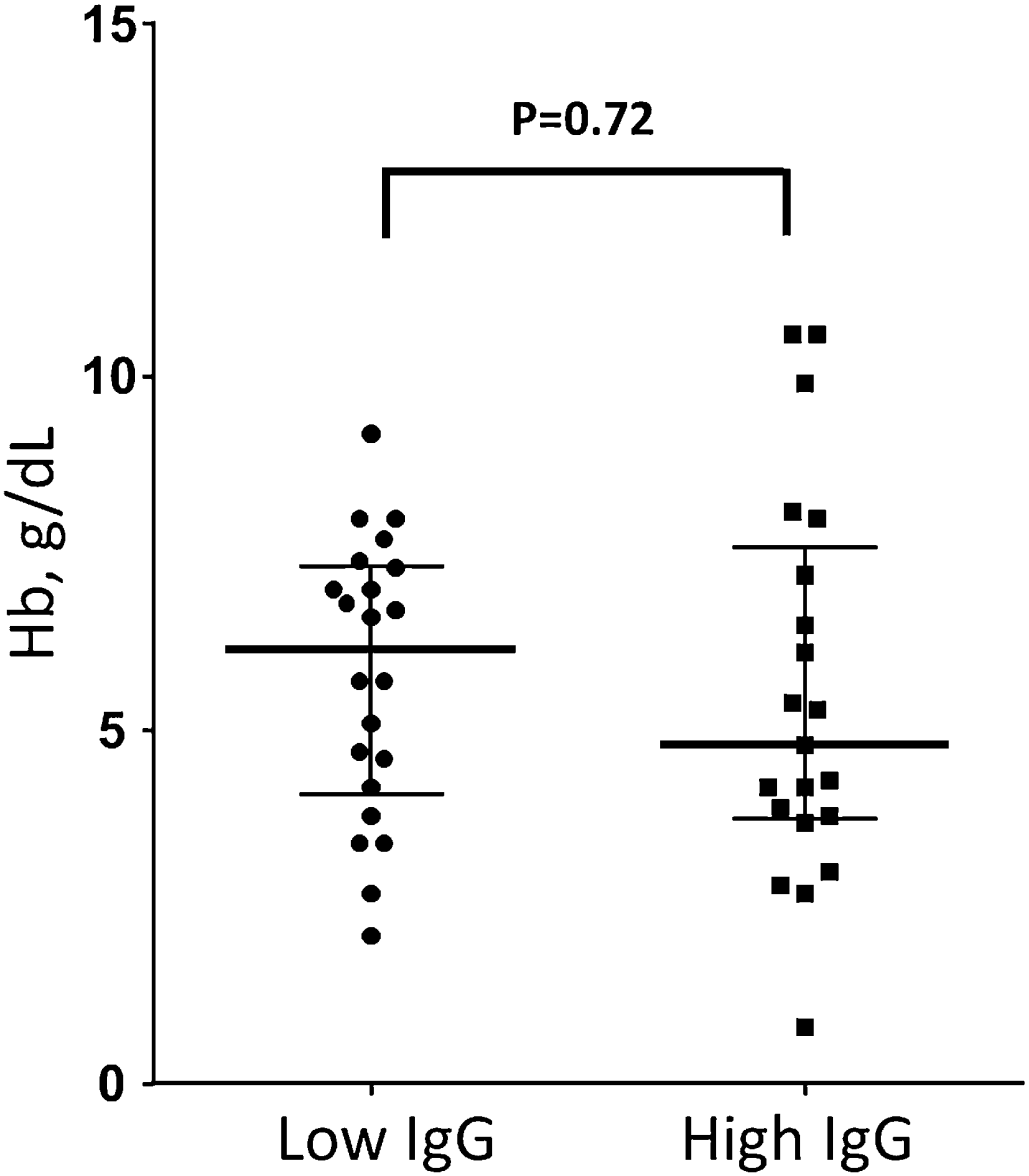
Hb levels in CM children at admission stratified by high (above median) and low (below median) IgG levels against AMA-1. Red bars indicate medians and interquartile range. Data points in both sets distributed normally. P value by unpaired t-test.

We found that individual children with CM at admission reacted variably to the PF11_0521 DBLβ3_D4 domain. When we stratified data by high and low reactivity relative to the median value, children group with low (below median) IgG reactivity against PF11_0521 DBLβ3_D4 domain had higher parasitemia (PE) (44,000 vs 12,000 parasites per microliter, not statistically significant) and lower Hb (P = 0.015 by t-test, as both groups of data were distributed normally) compared to group with high (above median) IgG. Median levels for Hb concentrations in these two groups were 4.65 (low IgG) and 6.5 g/dL (high IgG), respectively (Fig. 5). Not only this is a substantial difference of ~ 2 g/dL, but also median level for low IgG group is below severe anemia threshold (5 g/dL). Similarly, these plasmas reacted against the AMA-1 construct but associations with parasitemia and Hb levels were reversed to what was observed with the PF11_0521 DBLβ 3_D4 domain and not statistically significant (Fig. 6).

## Discussion

The feasibility of a protecting vaccine against malaria is supported by the naturally acquired immunity in people living in endemic areas. It has been shown that as the rate of exposure increase so does the level of protection against severe life-threatening malaria. This observation is underpinned by several immuno-epidemiological studies^30,31^ in which PfEMP1s and other VSAs antigens are important markers^10,32–34^. Here, we assessed the IgG antibody response in children with uncomplicated and severe cerebral malaria to investigate the onset of IgG response to the PfEMP1 protein PF3D7_1150400/PF11_0521. This PfEMP1 protein contains two remarkable features in its structure: (a) domain cassette DC13, which is composed of tandem of domains DBLα1.7 and CIDRα1.4, and binds EPCR receptor through CIDRα1.4^35^, and (b) neighboring downstream domain DBLβ3_D4, which binds ICAM-1 receptor^36^. It has been shown previously that the presence of these two features together in one protein plays a pivotal role in the pathogenesis of CM^19^. Therefore, we focused our efforts to measure IgG response against the entire DC13, functionally active (ICAM-1 binding) full-length domain DBLβ 3_D4, and smaller peptides located on the surface of these two constructs.

Our results show acquisition of IgG response against the entire DC13, which was stronger after convalescence than at admission with a difference in IgG1 and IgG3 level statistically significant between day 0 and day 30 in children with CM (Fig. 1 B). This outcome is in agreement with the specific expression of DC13 in parasites infecting children with severe and cerebral malaria^16,17^ thus indicating the acquisition of specific antibody response against this cassette. However, this response was also found in children with UM, which may indicate previous exposure of these children to epitopes from DBLα1.7 and CIDRα1.4, or sufficiently homologous domains. This difference between the response at admission and at day 30 was statistically significant (P = 0.001) (Fig. 1A) suggesting a boost in the antibody acquisition. This statement can be supported by the history of previous severe malaria episodes in children with UM, which reached 20% in our cohort. As the volume of plasma samples from children with uncomplicated malaria available to us was limited, these samples were not included in all experiments described below, which prevent us from making conclusions on UM group response in some of the experiments. This is one of the limitations of this study.

The IgG response against the DBLα1 A1-31 peptide located in DBLα1.7_D2 domain was also higher at convalescence than at admission in CM children (P < 0.0001) and was higher in semi-immune children (Fig. 1C). However, for another peptide (DBLα1 A2–42) located in DBLα 1.7_D2, IgG response was low and almost similar for admission day 0 and convalescence day 30. In addition, for this peptide a low prevalence of responders among semi-immune children was noticed (Fig. 1D). These results suggest that DBLα1 A1–31 peptide is more immunogenic and/or more conserved in the field isolates than peptide DBLα1 A2–42. Interesting that these peptides differ in their secondary structures in the domain used as a 3D model (PDB 2XU0) for mapping of these peptides. While DBLα1 A2-42 peptide is mapped to the two neighboring alpha helices, more reactive peptide DBLα1 A1-31 is mapped to the more disordered structure (Supplementary Fig. 3).

As, in this work, we assessed only responses to entire DC13 and to peptides located in DC13 domain DBLα1.7, further experiments to assess IgG response to CIDRα1.4 domain/peptides will expand current information on the development of specific response in children with CM and UM to DC13 cassette. This is another limitation of this study.

Our results on acquisition of IgG response to PF11_0521 DBLβ3_D4 (against peptides and entre domain) further contribute to understanding protective immune response to PfEMP1 proteins with dual receptor binding, important for CM.

With the exception of DBLβ3 B1-17 peptide, IgG response against other DBLβ3 peptides located in the DBLβ3-D4 domain was substantially different at admission and convalescence, with statistical significance for DBLβ3 B2-37 and DBLβ3 B3-34 in children with CM (Figs. 2 and 3). Furthermore, in children with UM this difference was also significant for peptide DBLβ3 B3-34 and the level of IgG1 was significantly higher in UM than CM at Day 30, in agreement with the hypothesis that group with UM is more protected than group with CM, and that antibodies against PfEMP1 antigens are likely to play an important role in the protection.

Peptides DBLβ3 B1-17, DBLβ3 B2-37, and DBLβ3 B3-34 are located in the PF11_0521 DBLβ3_D4, an ICAM-1 DBLβ binder^22^ which consist of a core of α helices connected by several loops^19^. Peptide DBLβ3 B1-17 is a part of two α helices and a loop, the DBLβ 3 B2-37 peptide constitute an entire α helix with two loops, while DBLβ3 B3-34 peptide is a helix containing residues essential for the direct interaction with ICAM-1, as well as a structural motif GGP involved in positioning of these residues^19^. The antibody response against this peptide was higher than against DBLβ3 B1-17 and DBLβ3 B2-37, with a high level of IgG1 in children with both UM and CM, as well as the AM group (Fig. 3B,C) suggesting a potentially protective role for these antibodies. This peptide seems to be highly immunogenic and probably conserved in the field parasites.

As we consistently see accumulation of antibody responses against DC13 and peptides located in both DBLα1.7_D2 and DBLβ3_D4 domains at convalescence (day 30) compared to admission (day 0), we tested for reactivity of these plasma samples against peptides from MSA antigen (Fig. 4A–C), which often serves to measure a level of exposure to malaria parasites. We found that for two MSA peptides, there is no difference in groups of children with CM at day 0 and day 30 (Fig. 4B,C), and for one peptide, there is (Fig. 4C). Thus, it is hard to make a definitive conclusion about exposure based on peptides reactivity. To overcome this problem, we used extracellular part (520 amino acid residues between signal sequence and trans-membrane domain) of AMA-1 protein as a control for exposure in groups of children with CM collected at days 0 and 30. Figure 4D demonstrates that exposure for these two groups is similar.

The reactivity of COS-7-expressed entire functional (ICAM-1 binding) PF11_0521 DBLβ3_D4 domain (immobilized on BioPlex beads) with plasma from children with CM at admission indicate that children with high IgG levels have higher Hb (P = 0.0159) and lower parasitemia (not statistically significant). This suggests that high levels of IgG against this domain are associated with protection of patients, which develop CM, against low Hb levels and severe anemia. As association was found at the admission, it indicates that IgG reactivity against this domain either persists after previous experience or quickly raised from immunological memory upon current infection, which leads to CM and hospitalization. If level is high enough, it may protect against severe anemia in these patients with CM (conjectured from the observed association). This observation is of high interest since severe malaria anemia is highly prevalent among children with severe malaria including CM and, in our cohort, it reaches 40%.

Interestingly, IgG response in children at admission and at day 30 is strong against the entire DC13 cassette, as well as against peptides DBLα1 A1-31 and DBLβ3 B3-34. For the DBLα1 A1-31 and DBLβ3 B3-34 peptides the level of this response was similar to that obtained in AM group, which consisted of semi-immune children from Cameroon, and in positive controls obtained from adults living in Senegal, suggesting the acquisition of IgG response to these antigens and potential role in protection.

These data also indicate that these peptides most likely represent conserved epitopes. Our results also indicate that IgG response is highly heterogeneous within patients in the same group, particularly in children with CM at day 30. This was even more noticeable for the IgG3 response against PF11_0521 DC13 (Fig. 1B). This difference in the antibody response within the group was not associated with hemoglobin or parasitemia for IgG3 and IgG1 against PF11_0521 DC13 (data not shown). We also noticed a significant positive correlation between the level of hemoglobin and that of IgG response to peptides DBLβ3 B1-17, DBLβ3 B2-37 and MSA3 C-27 in children with CM at admission, as well as statistically non-significant negative correlation between the level of IgG response and parasitemia (data not shown). These results are consistent with those obtained when measuring the IgG reactivity to the full-length ICAM-1-binding domain DBLβ3_D4 as presented in Fig. 5, and concur with previous findings in Papua New Guinea cohort^24^. Definitely, the non-statistically significant observation about difference in parasitemia in children with high and low IgG levels to PF11_0521 DBLβ3_D4, and peptides DBLβ3 B1-17, DBLβ3 B2-37 needs to be further confirmed. It can be done, for example, by using HRP2 ELISA-based parasite load quantification, which also accounts for the sequestered parasites. Besides, the heterogeneous response between individuals might also originate from the genetic polymorphism of genes associated with immune response, such as FcγRIIa and TNFα^37^. These hypotheses should be tested using larger set of samples.

The IgG1 and IgG3 isotypes are the cytophilic antibodies shown to be dominant in clinically protected compared to non-protected individuals, thus associated with acquired protective immunity^38–40^. Our results demonstrate an acquisition of IgG1 and IgG3 antibodies against entire PF11_0521 DC13 and against peptides from DBLβ3_D4 domain in a group of children with CM with statistically significant difference between admission and convalescence. However, for IgG1 and IgG3 responses in children with UM there was no difference between admission and convalescence, suggesting an existing immunity, which might be acquired due to previous exposure to these or homologous antigens. The IgG1 and IgG3 response was significantly higher in AM group than in CM and UM groups at day 30 highlighting the importance of these IgG isotypes in the immune protection.

Our results further support the idea of development of a vaccine based on PfEMP1 DBLβ domains that would elicit immune response against this domain and potentially prevent interactions with ICAM-1. However, other experiments to investigate the antibody-dependent cell-mediated cytotoxicity initiated by antibodies against purified DC13 as well as against DBLα1 A1-31, and DBLβ3 B3-34 peptides are needed to better comprehend relevance of these antigens as vaccine candidates. Also, additional studies of ICAM-1 binding inhibition activity using IgG from these plasmas will allow better understanding the development of immune protection against CM. It is also essential to assess the levels of IgG responses to these antigens in cohorts from other epidemiological settings.

## Supplementary Information

Supplementary Information 1.

Supplementary Information 2.

Supplementary Information 3.

Supplementary Information 4.

Supplementary Information 5.

Supplementary Information 6.

Supplementary Information 7.

Supplementary Information 8.

Supplementary Information 9.

Supplementary Information 10.

Supplementary Information 11.

Supplementary Information 12.
