# Author Correction: IgG acquisition against PfEMP1 PF11_0521 domain cassette DC13, DBLβ3_D4 domain, and peptides located within these constructs in children with cerebral malaria

**DOI:** 10.1038/s41598-021-89079-6

**Published:** 2021-04-29

**Authors:** Cyril Badaut, Pimnitah Visitdesotrakul, Aurélie Chabry, Pascal Bigey, Bernard Tornyigah, Jocelyne Roman, Jules Alao Maroufou, Annick Amoussou, Blaise Serge Ayivi, Gratien Sagbo, Nicaise Tuikue Ndam, Andrew V. Oleinikov, Rachida Tahar

**Affiliations:** 1 Institut de Recherche Biomédicale des Armées, National Reference Laboratory for Arboviruses, Marseille, France; 2 Charles E. Schmidt College of Medicine, Florida Atlantic University, Boca Raton, FL 33428 USA; 3 Université de Paris, MERIT, IRD, 75006 Paris, France; 4 Université de Paris, UMR 8151 CNRS - INSERM U1022 - ENSCP, 75006 Paris, France; 5 Département de Pédiatrie, Hôpital Mère-Enfant La Lagune (CHUMEL) Cotonou, Cotonou, Benin; 6 Service de Pédiatrie, Centre Hospitalo-Universitaire, Suruléré (CHU-Suruléré, Cotonou, Benin; 7 Service de Pédiatrie, Centre National Hospitalo-Universitaire (CNHU), Cotonou, Benin; 8 Institut de Recherche Pour le Développement (IRD), UMR 261 Mère et Enfant Face Aux Infections Tropicales, Université Paris-Descartes, 4, Avenue de l’observatoire, 75270 Paris, France

 Correction to: *Scientific Reports* 10.1038/s41598-021-82444-5, published online 11 February 2021

The original version of this Article contained an error in the spelling of the author Cyril Badaut which was incorrectly given as Badaut Cyril.

This error has now been corrected in the PDF and HTML versions of the Article.

