# Supplementary material for: IgG acquisition against PfEMP1 PF11_0521 domain cassette DC13, DBLβ3_D4 domain, and peptides located within these constructs in children with cerebral malaria
Source: Sci Rep. 2021 Feb 11;11:3680. doi: 10.1038/s41598-021-82444-5 (PMC7878510; doi:10.1038/s41598-021-82444-5)
Supplement: Supplementary file 1 — Supplementary Information 1. [file 41598_2021_82444_MOESM1_ESM.docx]

**Supplementary Material**

**Title:**

**IgG acquisition against PfEMP1 PF11_0521 domain cassette DC13, DBLβ3_D4 domain, and peptides located within these constructs in children with cerebral malaria**

Cyril, Badaut ^1^, Pimnitah Visitdesotrakul ^2^, Aurélie Chabry ^3^; Pascal Bigey ^4^, Bernard Tornyigah^3^, Jocelyne Roman ^3^, Maroufou J.Alao ^5^, Amoussou Annick ^6^ , Ayivi Serge^7^, Gratien Sagbo ^7^, Nicaise Tuikue Ndam^3^ ^,^, Andrew V. Oleinikov^2^, Rachida Tahar ^3^ *

^1^ Unité de Biothérapies anti-Infectieuses et Immunité, Institut de Recherche Biomédicale des Armées, 1 place du Général Valérie André BP73, 91223 Brétigny-sur-Orge Cedex – France

^2^ Charles E. Schmidt College of Medicine, Florida Atlantic University, Boca Raton, FL 33428, USA

^3^ Université de Paris, MERIT, IRD, F-75006 Paris, France

^4^ Université de Paris, UMR 8151 CNRS – INSERM U1022 – ENSCP ; F-75006 Paris, France

^5^ Département de pédiatrie, Hôpital Mère-enfant la lagune (CHUMEL) Cotonou, Bénin

^6^ Service de pédiatrie, Centre Hospitalo-Universitaire, Suruléré (CHU-Suruléré) Cotonou, Bénin

^7^ Service de pédiatrie, Centre National Hospitalo-Universitaire (CNHU), Cotonou, Bénin

Corresponding author: Rachida Tahar*

Institut de Recherche pour le Développement (IRD), UMR 261 Mère et enfant face aux infections tropicales, Université Paris-Descartes, 4, Avenue de l’observatoire, 75270 Paris

Email, [Rachida.Tahar@ird.fr](mailto:Rachida.Tahar@ird.fr)

Tel : 331 53 73 99 33

Fax : 331 53 73 96 17

Cell :233545440654

WhatsApp : 33637343053

**Supplementary Figure 1**

Alignments of the XP_001348176 PfEMP1/ PF3D7_ 1150400/ PF11_0521 domains DBLα1 (a) and DBLβ3 (b) to other relevant sequences. Conserved and variable sequences are highlighted: green: 100% similar; khaki: 80 to 100% similar; orange: 60 to 80% similar; gray: less than 60% similar.

**Supplementary Figure 2**

Alignment of peptides used in this work with the most homologous sequences of other DBL domains selected from alignments shown in Supplementary Figure 1: (a) DBLα A1-31 located between 24-55 and (b) DBLα A2-42 located between 137-185 of the DBLα1; (c) DBLβ3 B1-17 located between 178-204, DBLβ3 B2-37 located between 226-260 of the DBLβ3, (d) DBLβ3 B3-34 located between 388-420 of the DBLβ3.

**Supplementary Figure 3**

Sequential organization of domains in the PfEMP1 protein PF11_0521 and their known binding receptor specificity (top). Peptides selected for testing immune reactivity are shown in the 3D structure of their relevant domains: DBLα1 A1-31 located between 24-55 and DBLα1 A2-42 located between 137-185 of the DBLα1 domain (on the right); DBLβ3 B1-17 located between 178-204, DBLβ3 B2-37 located between 226-260 of the DBLβ3, DBLβ3 B3-34 located between 388-420 of the DBLβ3 domain (on the left).

**Supplementary Figure 4**. PF11_0521 DBLβ3_D4 domain immobilized on BioPlex beads is correctly folded as it has functional activity in binding ICAM-1 receptor. AFU, arbitrary fluorescence units. Error bars represent standard deviations.

**Supplementary material table 1:** Various peptides where selected from DBLs: (a) DBLα A1-31 located between 24-55 and (b) DBLα A2-42 located between 137-185 of the DBLα1, (c) DBLβ3 B1-17 located between 178-204, (d) DBLβ3 B2-37 located between 226-260 and (e) DBLβ3 B3-34 located between 388-420 of the DBLβ3.

**Supplementary material table 2:** The median reactivity and 25%-75% interquartile ranges for each the DC13 and peptides.
