# Supplementary material for: IgG acquisition against PfEMP1 PF11_0521 domain cassette DC13, DBLβ3_D4 domain, and peptides located within these constructs in children with cerebral malaria
Source: Sci Rep. 2021 Feb 11;11:3680. doi: 10.1038/s41598-021-82444-5 (PMC7878510; doi:10.1038/s41598-021-82444-5)
Supplement: Supplementary file 2 — Supplementary Information 2. [file 41598_2021_82444_MOESM2_ESM.docx]

| **Supplementary Table 1** |  |  |  |
| --- | --- | --- | --- |

| Name | Sequence | Lenght (aa) | Domain | Location |
| --- | --- | --- | --- | --- |
| DBLα1 A1-31 | AGRNRNRFSYEGEAECRISRITGNKTEHG | 31 | DBLα1.7 | 98-127 $ |
| DBLα1 A2-42 | LGNGDYKEKVSNNLRAIFNKIYENLNDPKLKKHYQKDAPN | 42 | DBLα1.7 | 210-248 $ |
| DBLβ3 B2-17 | TMKDYKSKNDKEGI | 17 | DBLβ3 | 884-898 * |
| DBLβ3 B3-37 | KDGGEIKTQNHLVTIFDKIKAQLPKDIKGKYTG | 37 | DBLβ3 | 920-956 * |
| DBLβ3 B3-34 | WDAISYKYLMLYAKARITAINGGPGYYNTEVQ | 34 | DBLβ3 | 1062-1093* |

*relative to ﻿PF11_0521 PFEMP1 (PDB 5mza)

$ relative to (PDB 2XU0)
