# Supplementary material for: IgG acquisition against PfEMP1 PF11_0521 domain cassette DC13, DBLβ3_D4 domain, and peptides located within these constructs in children with cerebral malaria
Source: Sci Rep. 2021 Feb 11;11:3680. doi: 10.1038/s41598-021-82444-5 (PMC7878510; doi:10.1038/s41598-021-82444-5)
Supplement: Supplementary file 3 — Supplementary Information 3. [file 41598_2021_82444_MOESM3_ESM.docx]

|  | Protein/ Peptides | | | | | |  | |
| --- | --- | --- | --- | --- | --- | --- | --- | --- |
| Children group | Pf 11-0521 DC13 | DBLα1_ A1-31 | DBLα1 A2-42 | DBLβ3 B1-17 | DBLβ3 B2-37 | DBLβ3 B3-34 | |  |
| CM D0 IgG | 1.21 (1.07-1.35) | 1.64 (1.44-2.19) | 1.15 (0.92-1.43) | 1.22(1.08-1.36) | 1.06(0.99-1.33) | 2.27 (1.89-2.89) | |  |
| CM D0 IgG1 | 1.64 (1.43-1.86) |  |  |  |  | 1.26 (0.91-1.68) | |  |
| CM D0 IgG3 | 1.63 (1.14-2.12) |  |  |  |  | 1.89 (1.58-2.49) | |  |
| CM D30 IgG | 1.34 (1.11-1.46) | 3.07 (2.47-3.71) | 1.10 (0.98-1.30) | 1.25 1.07-1.55) | 1.45 (1.37-1.56) | 2.88 (2.43-3.34) | |  |
| CM D30 IgG1 | 2.29 (2.04-2.76) |  |  |  |  | 2.27 (1.89-2.89) | |  |
| CM D30 IgG3 | 5.91 (2.98-7.33) |  |  |  |  | 2.29 (1.54-2.94) | |  |
| UM D0 IgG | 1.06 (0.89-1.39) |  | 0.97 (0.83-1.04) | 1.22 (1.17-1.27) | 1.10 (1.01-1.17) | 2.17 (1.76-2.71) | |  |
| UM D0 IgG1 |  |  |  |  |  | 2.70 (2.27-3.32) | |  |
| UM D0 IgG3 |  |  |  |  |  | 1.66 (1.44-2.13) | |  |
| UM D30 IgG | 1.36 (1.10-1.68) |  | 1.17 (0.88-1.34) | 1.32 (1.20-1.77) | 1.14 (1.03-1.56) | 2.61 (2.23-3.22) | |  |
| UM D30 IgG1 |  |  |  |  |  | 2.74 (2.23-3.43) | |  |
| UM D30 IgG3 |  |  |  |  |  | 1.86 (1.76-2.72) | |  |
| AM IgG | 1.48 (1.36-1.63) | 2.21 (1.81-2.42) | 1.29 (1.12-1.58) | 1.59 (1.26-1.98) | 1.37 (1.12-1.65) | 2.74 (2.47-3.09) | |  |
| AM IgG1 |  |  |  |  |  | 3.21 (2.58-3.63) | |  |
| AM IgG3 |  |  |  |  |  | 3.05 (2.51-3.37) | |  |
|  |  |  |  |  |  |  | |  |
| Children group | MSA3 A-27 | MSA3 B-42 | MSA3 C-27 |  |  |  | |  |
| CM D0 IgG | 1.34 (1.16-1.62) | 1.44 (1.18-2.09) | 0.94 (0.76-1.81) |  |  |  | |  |
| CM D30 IgG | 1.34 (1.27-1.71) | 1.37 (1.14-2.05) | 2.1 (1.77-2.57) |  |  |  | |  |
| AM IgG | 2.69(1.61-3.09) | 2.15 (1.68-2.99) | 3.28 (2.68-3.86) |  |  |  | |  |
