# Supplementary figures and images for: IgG acquisition against PfEMP1 PF11_0521 domain cassette DC13, DBLβ3_D4 domain, and peptides located within these constructs in children with cerebral malaria
Source: Sci Rep. 2021 Feb 11;11:3680. doi: 10.1038/s41598-021-82444-5 (PMC7878510; doi:10.1038/s41598-021-82444-5)

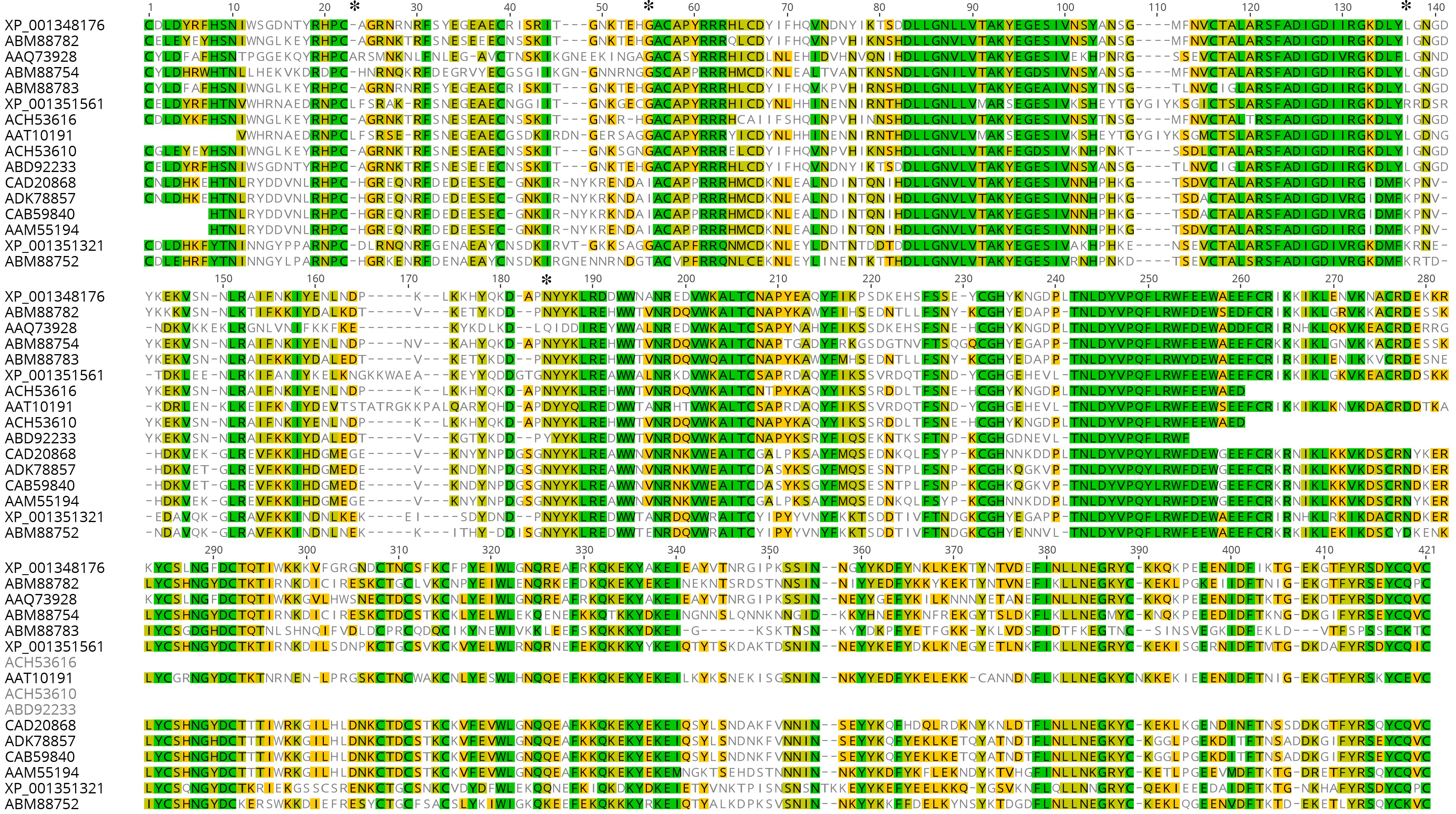

Supplement: Supplementary file 4 — Supplementary Information 4. [file 41598_2021_82444_MOESM4_ESM.jpg]

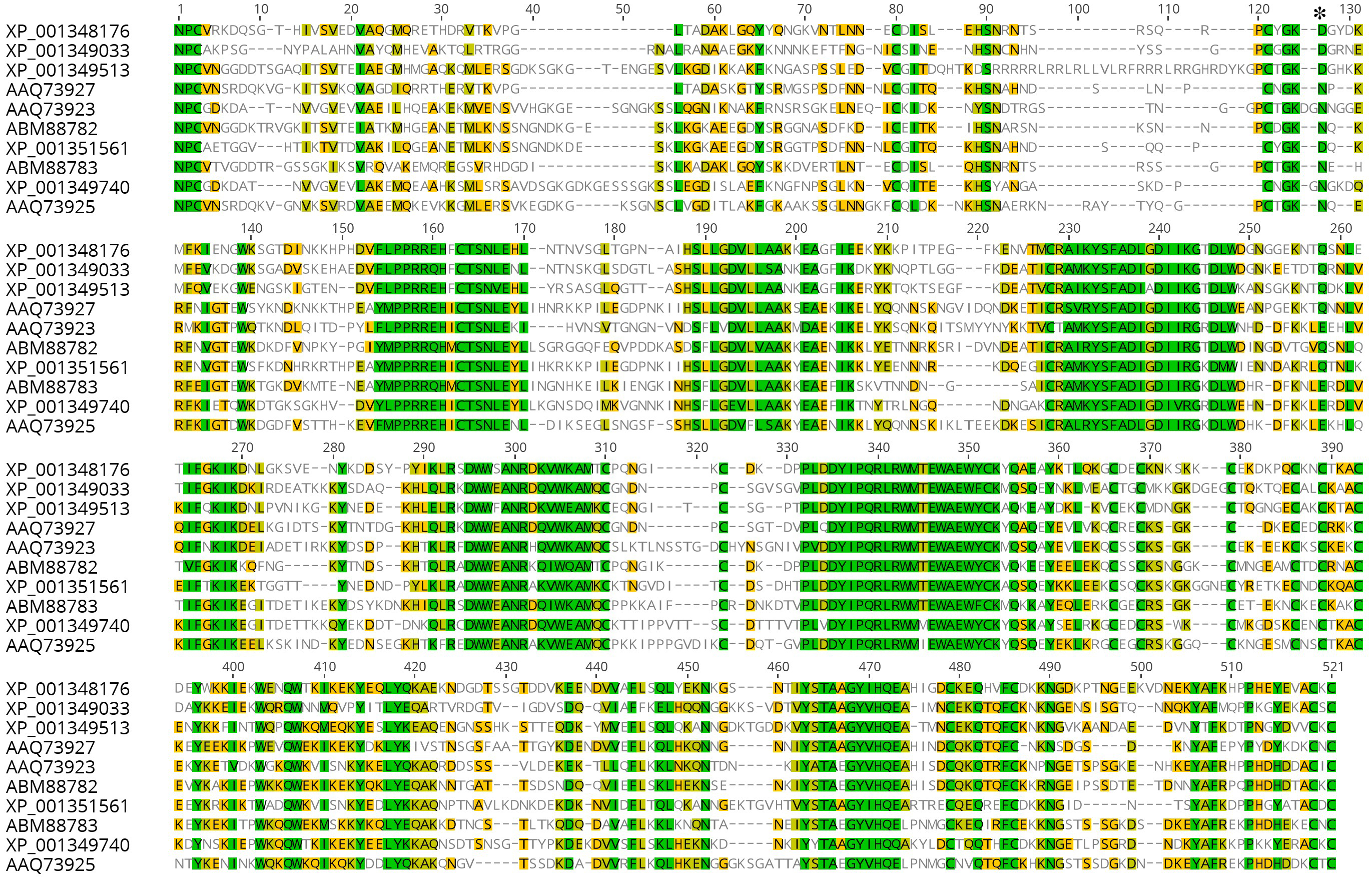

Supplement: Supplementary file 5 — Supplementary Information 5. [file 41598_2021_82444_MOESM5_ESM.jpg]

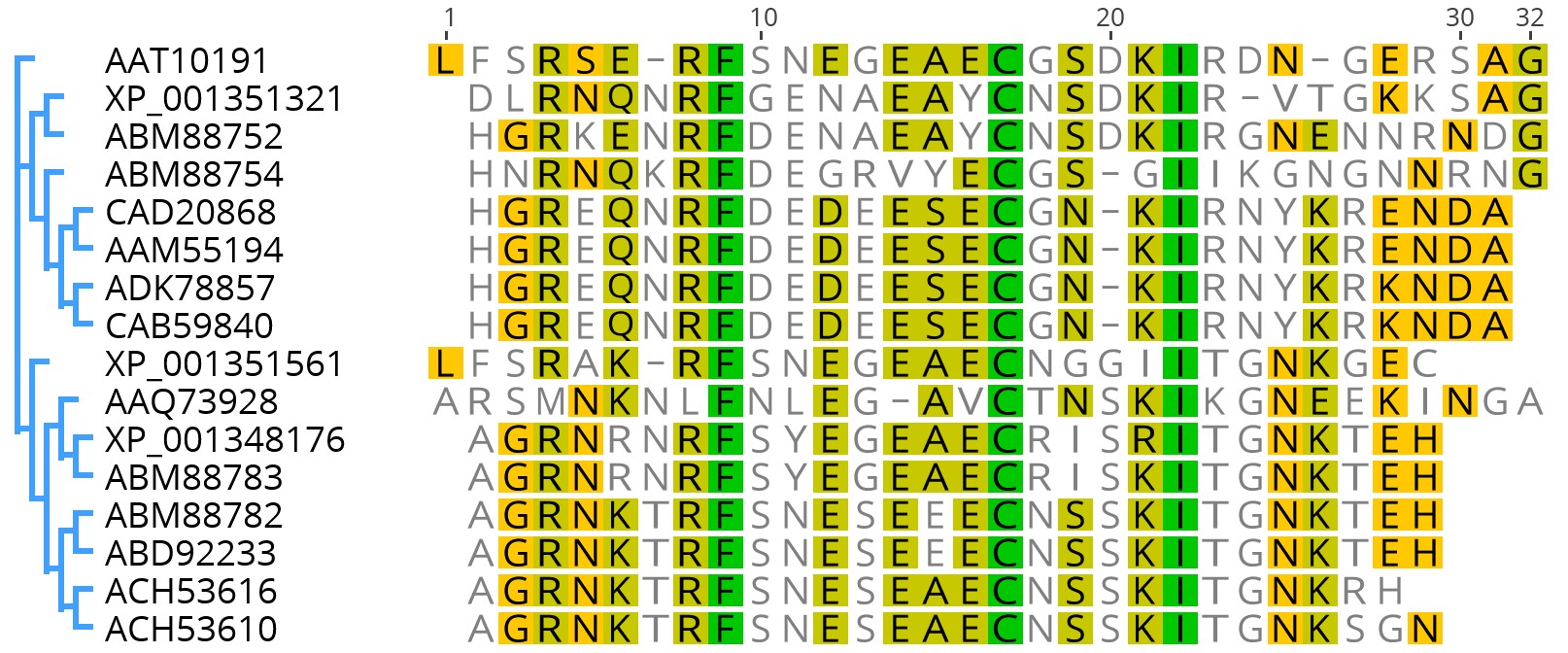

Supplement: Supplementary file 6 — Supplementary Information 6. [file 41598_2021_82444_MOESM6_ESM.jpg]

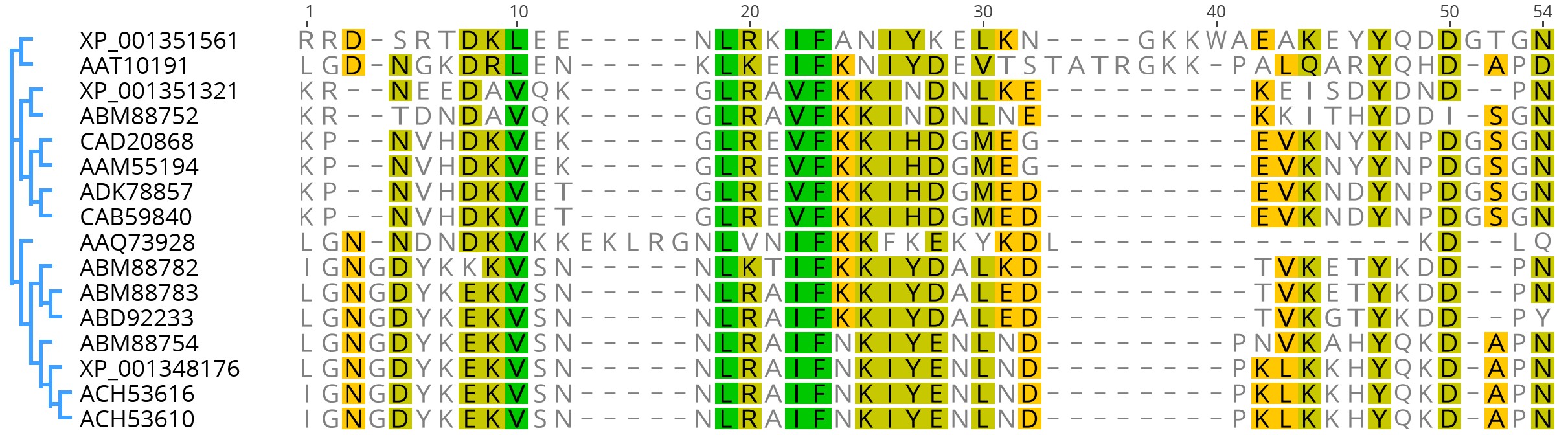

Supplement: Supplementary file 7 — Supplementary Information 7. [file 41598_2021_82444_MOESM7_ESM.jpg]

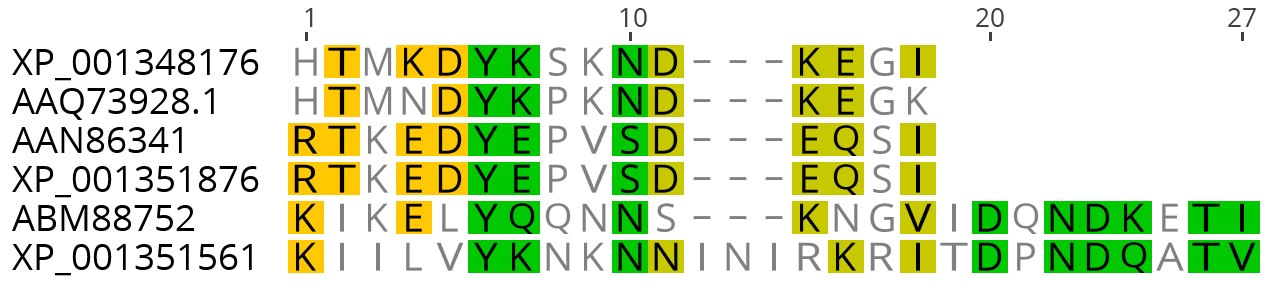

Supplement: Supplementary file 8 — Supplementary Information 8. [file 41598_2021_82444_MOESM8_ESM.jpg]

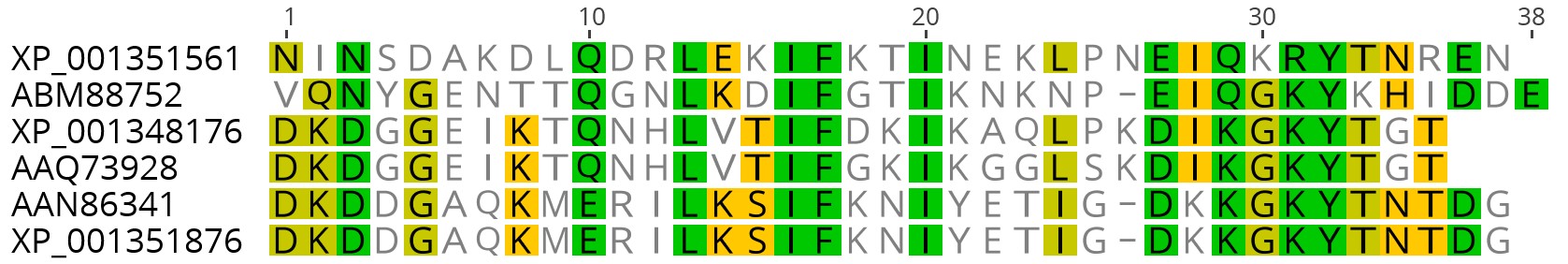

Supplement: Supplementary file 9 — Supplementary Information 9. [file 41598_2021_82444_MOESM9_ESM.jpg]

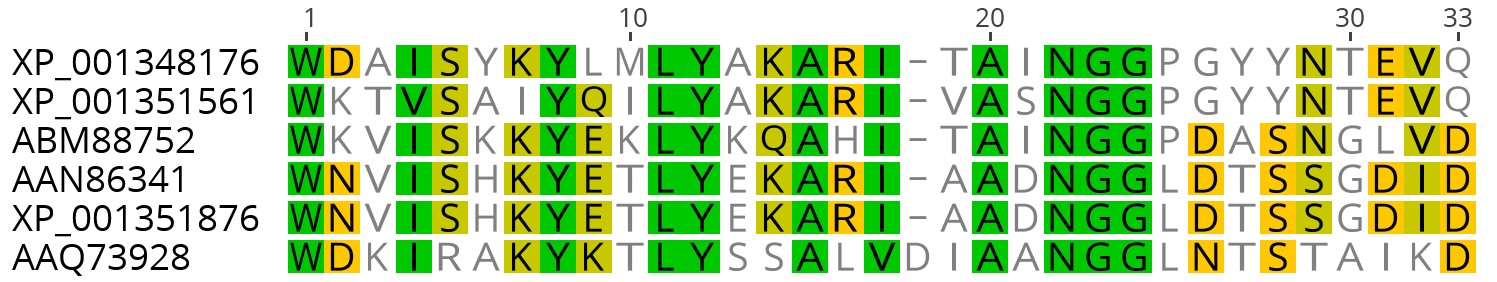

Supplement: Supplementary file 10 — Supplementary Information 10. [file 41598_2021_82444_MOESM10_ESM.jpg]

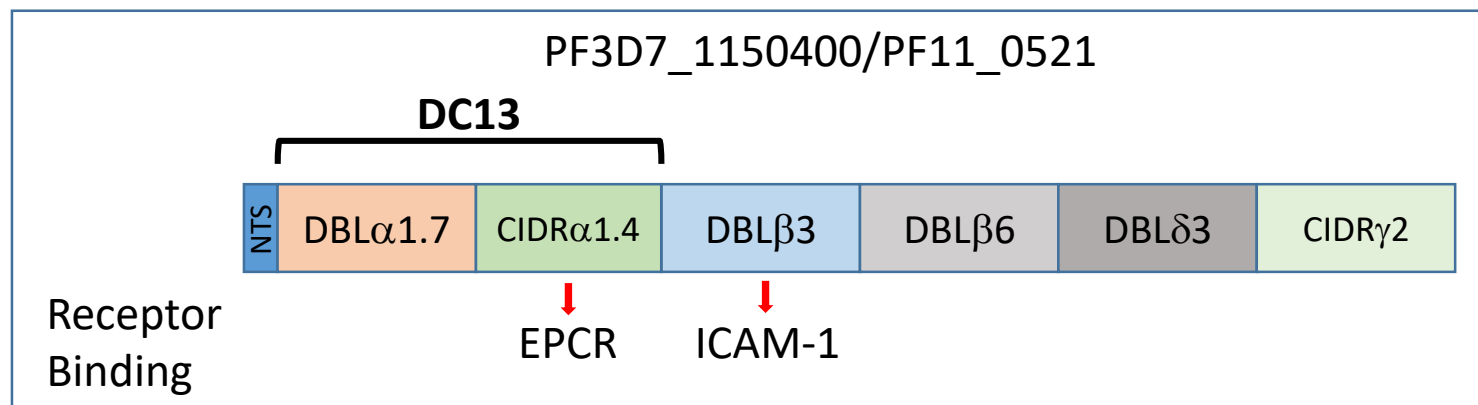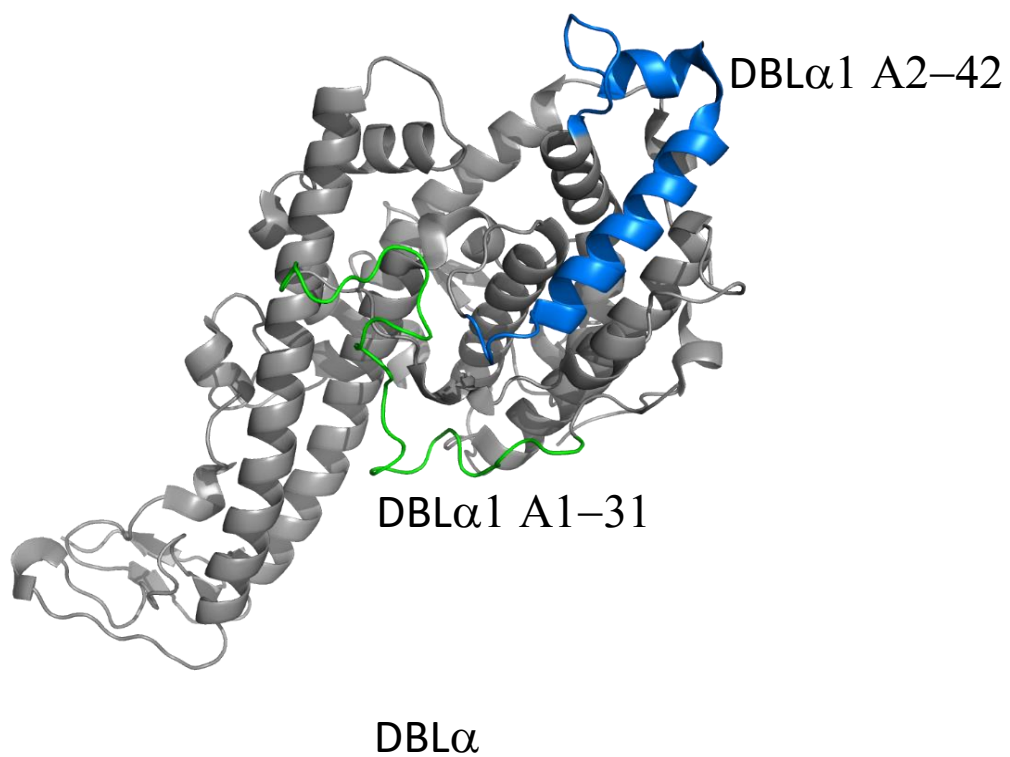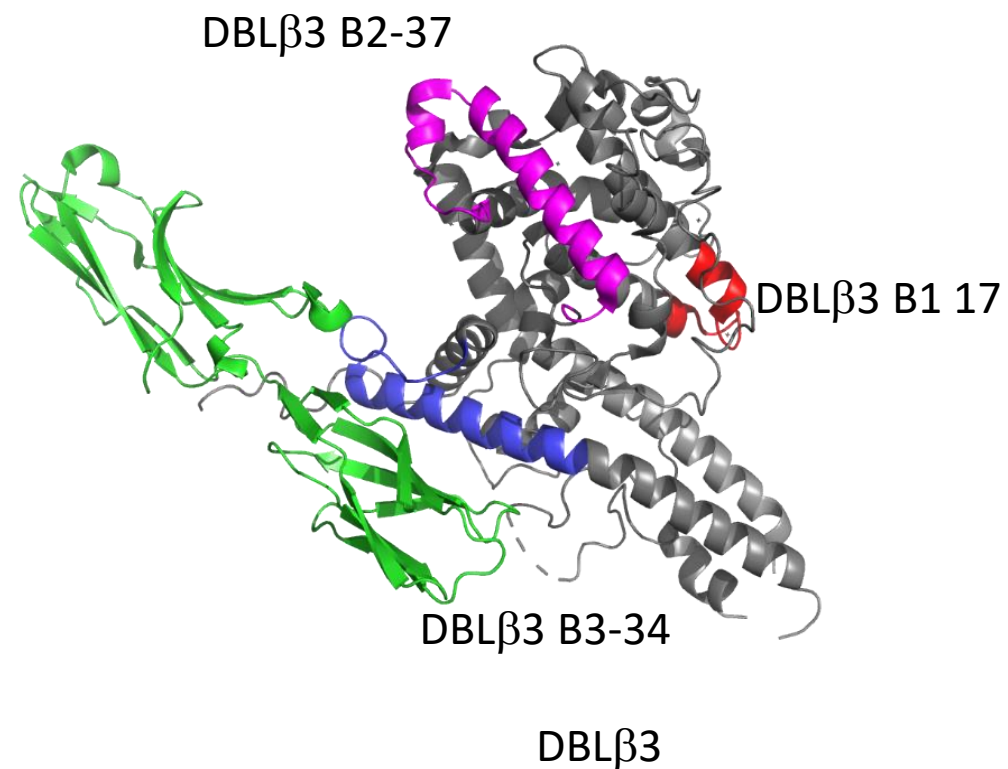

Supplement: Supplementary file 11 — Supplementary Information 11. [file 41598_2021_82444_MOESM11_ESM.pdf]
