# Supplementary material for: IgG acquisition against PfEMP1 PF11_0521 domain cassette DC13, DBLβ3_D4 domain, and peptides located within these constructs in children with cerebral malaria
Source: Sci Rep. 2021 Feb 11;11:3680. doi: 10.1038/s41598-021-82444-5 (PMC7878510; doi:10.1038/s41598-021-82444-5)
Supplement: Supplementary file 12 — Supplementary Information 12. [file 41598_2021_82444_MOESM12_ESM.pdf]

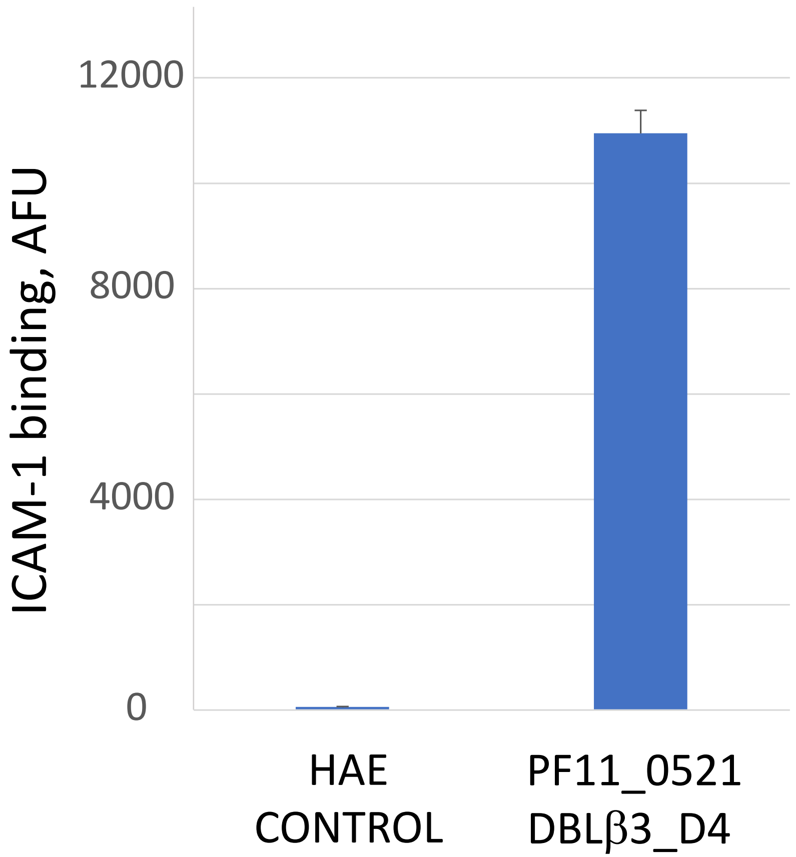

Supplementary Figure 4. PF11_0521 DBLβ3_D4 domain immobilized on BioPlex beads is correctly folded as it has functional activity in binding ICAM-1 receptor. AFU, arbitrary fluorescence units. Error bars represent standard deviations.
